# Genome Editing and the Future of Farming meeting report

**DOI:** 10.1007/s11248-016-0006-x

**Published:** 2017-01-31

**Authors:** Monica Hoyos-Flight, Emilie Brady, Helen Sang, Bruce Whitelaw

**Affiliations:** The Roslin Institute and Royal (Dick) School of Veterinary Studies, University of Edinburgh, Easter Bush Campus, Midlothian, EH25 9RG UK

On the 6th September 2016 leaders in the field of livestock genetics, regulation and food supply gathered at The Roslin Institute to discuss the latest technologies and their applications in a one day meeting co-sponsored by the OECD Co-operative Research Programme: Biological Resource Management for Sustainable Agricultural Systems, the UK’s National Institutes of Bioscience (NIB) and the Centre for Tropical Livestock Genetics and Health (CTLGH), a collaboration between the University of Edinburgh, Scotland’s Rural College and the International Livestock Research Institute.

“Genome Editing and the Future of Farming” was a free, open meeting that attracted over 100 representatives from a range of research organisations, industry, funding and policy-making bodies from across the globe. The meeting contributes to the ongoing dialogue among specialists from different backgrounds. As was pointed out in more than one occasion, this is instrumental for driving future agricultural innovation.

David Hume, Director of The Roslin Institute and Chair of NIB, introduced the first session in which Wayne Powell, Principal of Scotland’s Rural College, gave an overview of the global challenges faced by agriculture. As he pointed out, a new way of thinking will be crucial to deliver the predicted requirement for 70% more food by 2050 without destroying the environment. He highlighted the importance of global and regional trade systems as the global population balance changes, and the need for resilience in the food chain. The pursuit of scientific excellence and collaborations is key to meeting these daunting challenges.

Sequencing technology and informatics have revolutionised biology with major implications for agriculture. Genome editing techniques, which allow precise and quick modifications of plant and animal genes, are poised to build on this progress. Indeed, genome editing has already had a bigger public impact in a shorter period of time than Dolly the Sheep, argued Bruce Whitelaw, Head of the Developmental Biology Division at The Roslin Institute. In his presentation on the current state of technology, he brought everyone up to speed on the available genome editing tools that have been used successfully in pigs, cattle, chickens and sheep to improve their productivity and welfare. Pig 26 is an example that made headlines earlier this year as it carries a specific single-base deletion in a gene that may stop them from contracting African Swine Fever.

Next up was Jonathan Lightner from Genus plc. who spoke about the opportunities and challenges of genome editing for the animal breeding industry. He introduced some of the projects being carried out in collaboration with academic research organisations to tackle porcine reproductive and respiratory syndrome (by editing a key virus entry mediator CD163) and bovine respiratory disease complex (by editing a single amino acid in CD18 signal peptide). There was general consensus that despite the great potential of such projects, the current uncertainties about the evolving regulatory frameworks and public acceptance could delay these genome edited animals from reaching the market.

After lunch Helen Sang (The Roslin Institute) chaired a session in which four speakers presented examples of their work on genome editing in a range of species. Tim Doran, CSIRO, explained how editing avian primordial germ cells may enhance vaccine production in eggs and improve food safety by reducing levels of egg allergens such as ovomucoid. Bhanu Telugu, University of Maryland, has used genome editing in pigs to remove *NANOS*, a key gene for male-specific germline development. Such animals can serve as hosts for spermatogonial stem cell transplantation from elite boars and be used for breeding, thus accelerating the expansion of desirable traits. Next, Goetz Laible from AgResearch spoke about the opportunities to improve cow’s milk using genome editing technologies. Not only can the major allergenic protein beta-lactoglobulin be eliminated, but milk proteins can be humanized or tailored to suit specific nutritional requirements.

The final speaker in this session, Steven Kemp, CTLGH, highlighted the role of the environment in driving genetic diversity. Understanding the genetics underlying natural variation and applying the latest genome editing technologies could increase the rates of genetic gain in livestock in developing countries. CTLGH brings together researchers in Scotland and Africa to address the need to improve productivity and sustainability in livestock systems in tropical climates.

The meeting’s next two sessions were panel discussions in which the chairs encouraged delegate participation. The first session, chaired by Federica Di Palma (Director of Science, Earlham Institute), focussed on advances in genome annotation. The panel members: Wesley Warren (McDonnell Genome Institute at Washington University), Dirk-Jan De Koning (Swedish University of Agricultural Sciences), Alan Archibald and John Hickey (The Roslin Institute) gave short presentations on their work in this area before addressing questions from the chair and the audience.

Genome editing for genetic improvement does not just require accurate genome sequences, it relies on the identification of the locations of genes and regulatory elements in a genome, as well as knowledge about their function. New sequencing and mapping technologies are aiding the generation of such “genome manuals” but there was a general consensus that funding large collaborative projects such as functional annotation of animal genomes (FAANG) is vital if the available genome editing technologies are to reach their potential.

The panel also discussed the fact that many economically important traits in livestock are complex traits that result from variation within multiple genes and their interaction with behavioural and environmental factors. Although a large number of quantitative trait loci (QTL) affecting complex traits in livestock have been identified, finding and validating specific quantitative trait nucleotides (QTN) underlying the QTL remains challenging. To prioritise target variants, De Koning proposed focussing on genetic defects due to recessive lethal alleles. An alternative strategy being pursued by Hickey and his group is to sequence hundreds of thousands of animals at low coverage and to identify causal variants underlying polygenic traits. Both will contribute to the ‘trait discovery pipeline’ that is needed if genome editing technology is to have widespread utility in agriculture.

The second panel session was chaired by James Smith (Vice Principal International, University of Edinburgh) and focussed on the regulation of and public dialogue around genome editing. This session’s panellists: Elisabeth Waigmann (EFSA), Alison Van Eenennaam (University of California, Davis), Huw Jones (IBERS) and Laura Bellingan (Royal Society of Biology) had an opportunity to compare their experiences and speak about what they personally or their organisations are doing to address the disconnect between the technology and the policy frameworks that regulate it in different countries. At present, it is unclear whether genome edited animals will be regulated as GMOs. The consensus was that concerns about regulation are already stalling the development and application of (and investment in) the technology. However, even if genome edited plant and animal products are to be regulated on a case-by-case or category basis, consumers will need to be made aware of the benefits they offer. It is the ‘what’ and ‘why’ rather than the ‘how’ which is important in this debate. The panel agreed that now was a key time for researchers to get involved and contribute to framing the public debate, and that an ongoing, direct dialogue is paramount to gain public acceptance for good applications of the technology.

As Bruce Whitelaw drew the day to a close, he reinforced the message that genome editing is a game-changing technology and that Society, Systems (regulatory and funding) and Science (the 3Ss) have to work together to ensure that it can be developed and applied to achieve the sustainable productivity gains that global agriculture requires.

*The opinions expressed and arguments employed in this publication are the sole responsibility of the authors and do not necessarily reflect those of the OECD or of the governments of its Member countries.*

*The Roslin Institute receives strategic funding from the Biotechnology and Biological Sciences Research Council (BBSRC)*.

